# Dopaminergic denervation and associated MRI microstructural changes in the nigrostriatal projection in early Parkinson’s disease patients

**DOI:** 10.1038/s41531-023-00586-x

**Published:** 2023-10-19

**Authors:** M. López-Aguirre, M. Matarazzo, J. Blesa, M. H. G. Monje, R. Rodríguez-Rojas, A. Sánchez-Ferro, J. A. Obeso, J. A. Pineda-Pardo

**Affiliations:** 1 HM CINAC (Centro Integral de Neurociencias Abarca Campal). Hospital Universitario HM Puerta del Sur, HM Hospitales, Madrid, Spain; 2 PhD Program in Physics, Complutense University of Madrid, Madrid, Spain; 3 Centro de Investigación Biomédica en Red de Enfermedades Neurodegenerativas (CIBERNED), Instituto de Salud Carlos III, Madrid, Spain; 4 Aligning Science Across Parkinson’s (ASAP) Collaborative Research Network, Chevy Chase, MD 20815 USA; 5 Ken and Ruth Davee Department of Neurology, Northwestern University, Feinberg School of Medicine, Chicago, IL USA; 6 Department of Neurology, University Hospital 12 de Octubre, Madrid, Spain; 7 Department of Medicine, Complutense University of Madrid, Madrid, Spain; 8 University CEU-San Pablo, Madrid, Spain

**Keywords:** Diagnostic markers, Parkinson's disease

## Abstract

Loss of dopaminergic neurons in the substantia nigra pars compacta (SNc) and a profound reduction of striatal dopamine are two hallmarks of Parkinson’s disease (PD). However, it’s unclear whether degeneration starts at the neuronal soma or the striatal presynaptic terminals, and how microstructural degeneration is linked to dopaminergic loss is also uncertain. In this study, thirty de novo PD patients and twenty healthy subjects (HS) underwent 6-[^18^F]-fluoro-L-dopa (FDOPA) PET and MRI studies no later than 12 months from clinical diagnosis. FDOPA uptake rate (*K*_i_), fractional volume of free-water (FW), and iron-sensitive R2* relaxometry were quantified within nigrostriatal regions. Inter-group differences (PD vs HS) were studied using non-parametric statistics and complemented with Cohen’s *d* effect sizes and Bayesian statistics. Correlation analyses were performed exploring biomarker dependencies and their association with bradykinesia scores. PD patients exhibited a significant decline in nigrostriatal dopaminergic activity, being post-commissural putamen (−67%) and posterolateral SNc (−11.7%) the most affected subregions within striatum and SNc respectively. Microstructural alterations (FW) were restricted to the hemisphere corresponding to the most affected side and followed similar spatial gradients as FDOPA *K*_i_ (+20% in posterior putamen and +11% in posterolateral SNc). R2* revealed no relevant significant changes. FDOPA and FW were correlated within the posterolateral SNc, and clinical severity was associated with FDOPA *K*_i_ loss. The asymmetry between striatal and SNc changes for both dopaminergic depletion and microstructural degeneration biomarkers is consistent with a neurodegenerative process that begins in the striatal terminals before progressing toward the cell bodies in the SNc.

## Introduction

The onset of cardinal features (bradykinesia, rigidity, and resting tremor) of Parkinson’s disease (PD) is classically associated with a 60-80% dopaminergic denervation^1–3^ concurrent with ~50% death of dopaminergic neurons in the substantia nigra pars compacta (SNc)^4,5^. Striatal dopaminergic loss typically follows a posterior-to-anterior gradient, with the greatest deficit in the posterior part of the putamen contralateral to the clinically affected hemibody^1,6,7^. Moreover, the highly focal clinical presentation of parkinsonism, typically manifested first in the hands, suggests a focal somatotopic origin, probably within the sensorimotor putamen^6,7^, although no somatotopical organization has been recognized in the SNc^8,9^. However, despite growing evidence supporting a distal origin of the pathological process at the terminals^10–16^, a pattern of structural and metabolic changes within the nigrostriatal system supporting this hypothesis is still lacking.

Magnetic resonance imaging (MRI) allows the assessment of brain microstructural changes in vivo, particularly by diffusion-weighted imaging (DWI). Pasternak and colleagues used a diffusion bi-tensor model to introduce free-water (FW) imaging^17^, which quantifies the contribution of non-constrained water molecules to the MRI signal. This MRI metric has become a promising biomarker for the assessment of nigral pathology in PD and other neurodegenerative disorders^18–21^. In addition to FW imaging, iron mapping is another MRI-derived biomarker that may be a key to understanding PD nigrostriatal pathology and neuronal vulnerability^22,23^. Iron radicals play an important role in the synthesis and metabolism of dopamine^24^, but on the other hand, they also participate in multiple biochemical reactions associated with neurodegeneration^24–26^. Several studies have reported iron overloads in the SN of PD patients, suggesting that iron accumulation within the nigrostriatal system might constitute a potential vulnerability factor in the pathogenesis^27–29^. Iron contents can be assessed semi-quantitatively using MRI by exploiting the paramagnetic properties of this metal, which induces magnetic field inhomogeneities that reduce local transverse relaxation time (T2*). As a result, the intensity of the T2* MRI signal, or its inverse R2* (1/T2*), will be proportional to the iron load^30^.

Here we aimed to study structural and metabolic alterations within the nigrostriatal system at the clinical onset of motor features in a group of de novo PD patients. We assessed the degree and spatial pattern of dopaminergic denervation (6-[^18^F]-fluoro-L-dopa, a.k.a. FDOPA), microstructural integrity (FW), and iron accumulation (R2*) within several regions of the nigrostriatal system, including spatial divisions of the SNc and striatum. Further, we tested for a correlation between clinical impairment and the degree and topography of nigrostriatal alterations.

## Results

### Dopaminergic activity

**Fig. 1:**
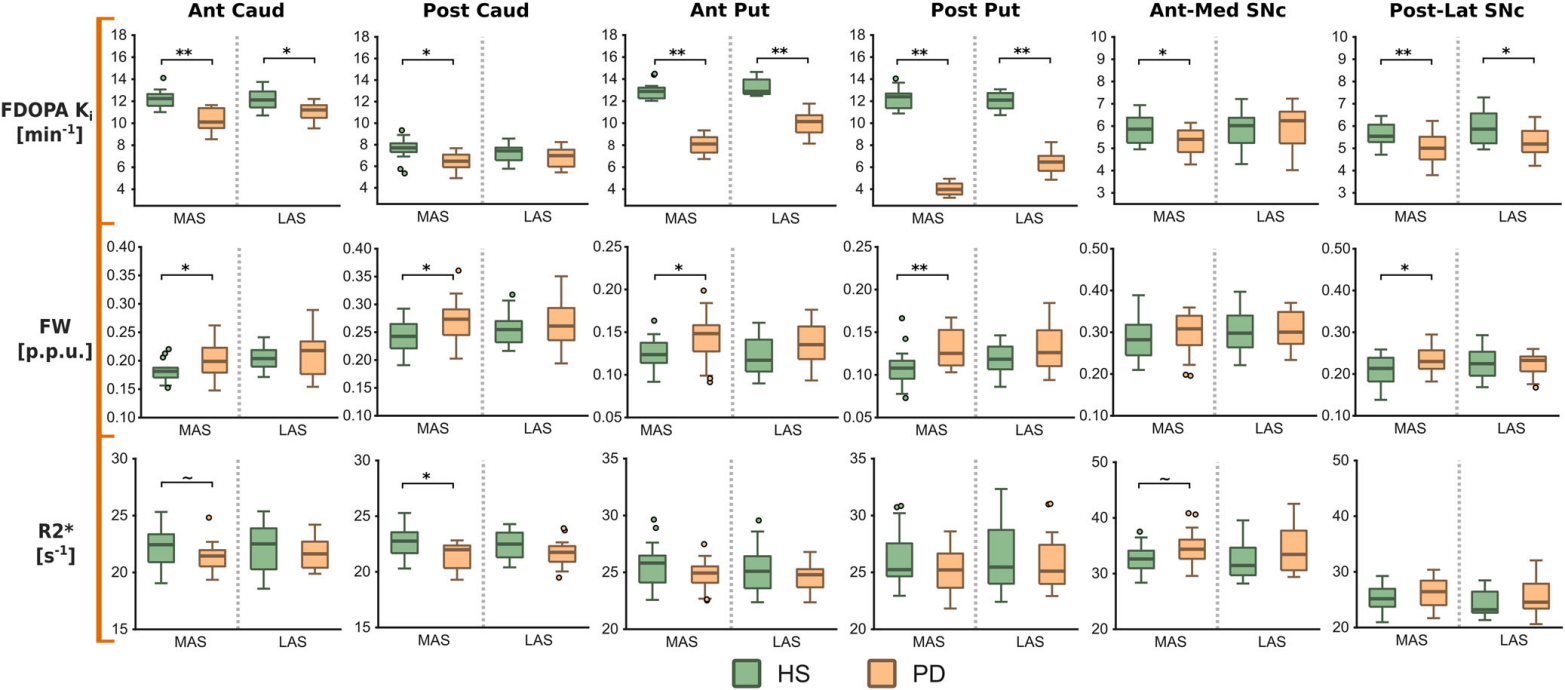
Inter-group comparison of the imaging metrics. Boxplot representation of the imaging metrics (FDOPA *K*_i_, FW, and R2*) comparing HS and PD groups, in green and orange colors respectively. Boxplots are grouped by more affected side (MAS) and dominant side, and by less affected side (LAS) and non-dominant side. Inter-group statistical significances after Mann–Whitney *U* tests are displayed above boxplots as *P* > 0.10 (no symbol); 0.10 ≤ *P* < 0.05 (~); 0.05 ≤ *P* < 0.01 (*); *P* ≤ 0.01 (**). In boxplots, the center line denotes the median, lower and upper box lines respectively represent the first and third quartiles, whiskers the minimum and maximum values, and points the outliers.

Striatal dopaminergic impairment followed a gradient of highest to lowest percentage FDOPA *K*_i_ reduction from the posterior putamen to the anterior putamen, the anterior caudate, and the posterior caudate (see Table 1 and Fig. 1). The putamen showed a marked FDOPA *K*_i_ decay, with the greater loss located in the posterior putamen (67% and 47% in more and less affected sides (MAS and LAS) respectively, d > 6.5, *P* < 0.01, *K* > 2). In the caudate both divisions displayed a significant FDOPA K_i_ decay in the MAS (15%, *d* > 1.3, *P* < 0.05, *K* > 2), whereas only the anterior division reached significance in the LAS (10%, *d* = 1.4, *P* < 0.05, *K* > 2). In the SNc, FDOPA *K*_i_ decline was close to 10% (*d* > 0.90, *P* < 0.05, *K* > 1) in the posterolateral division in both hemispheres and in the anteromedial division in the MAS (*d* = 0.90, *P* < 0.05, *K* > 1). See Supplementary Table 1 for a detailed listing of all FDOPA measures.

**Table 1:** Inter-group differences for F-Dopa *K*_i_, FW, and R2* maps.

		MAS vs DS			LAS vs nDS		
		*d*	*P*	*K*	*d*	*P*	*K*
FDOPA [min^−1^]	Ant Caud	−2.11	**	5.22	−1.40	*	2.91
	Post Caud	−1.34	*	2.95	−0.39	-	0
	Ant Put	−6.03	**	6.29	−3.57	**	6.31
	Post Put	−10.78	**	6.80	−6.53	**	5.95
	Ant-Med SNc	−0.90	*	1.48	0.07	-	0
	Post-Lat SNc	−1.03	**	1.99	−0.84	*	1.24
FW [p.p.u.]	Ant Caud	0.70	*	0.56	0.20	-	0
	Post Caud	0.80	*	0.98	0.17	-	0
	Ant Put	0.76	*	0.54	0.60	-	0.14
	Post Put	0.96	**	1.47	0.58	-	0
	Ant-Med SNc	0.22	−	0	0.03	-	0
	Post-Lat SNc	0.80	*	0.61	−0.06	-	0
R2* [s^−1^]	Ant Caud	−0.67	~	0.12	−0.33	-	0
	Post Caud	−0.98	*	1.49	−0.57	-	0
	Ant Put	−0.61	−	0	−0.40	-	0
	Post Put	−0.49	−	0	−0.20	-	0
	Ant-Med SNc	0.62	~	0.10	0.47	-	0.06
	Post-Lat SNc	0.37	−	0	0.40	-	0

Cohen effect size classification: *d* < 0.5 (small); 0.5 ≤ *d* < 0.8 (moderate); 0.8 ≤ *d* < 1.3 (large); *d* > 1.3 (very large). The sign of *d* represents the direction of the effect, i.e., for A vs B, *d* < 0 means that A < B and vice versa.

Significance of *P* values: *P* < 0.10 (−); 0.10 ≤ *P* < 0.05 (~); 0.05 ≤ *P* < 0.01 (*); *P* ≤ 0.01 (**).

Interpretation of BF [*K* = log10(BF)]: 0 < K ≤ 0.5 (barely worth mentioning); 0.5 < *K* ≤ 1 (substantial); 1 < *K* ≤ 2 (strong); *K* > 2 (decisive).

### MRI assessment of nigrostriatal integrity

**Fig. 2:**
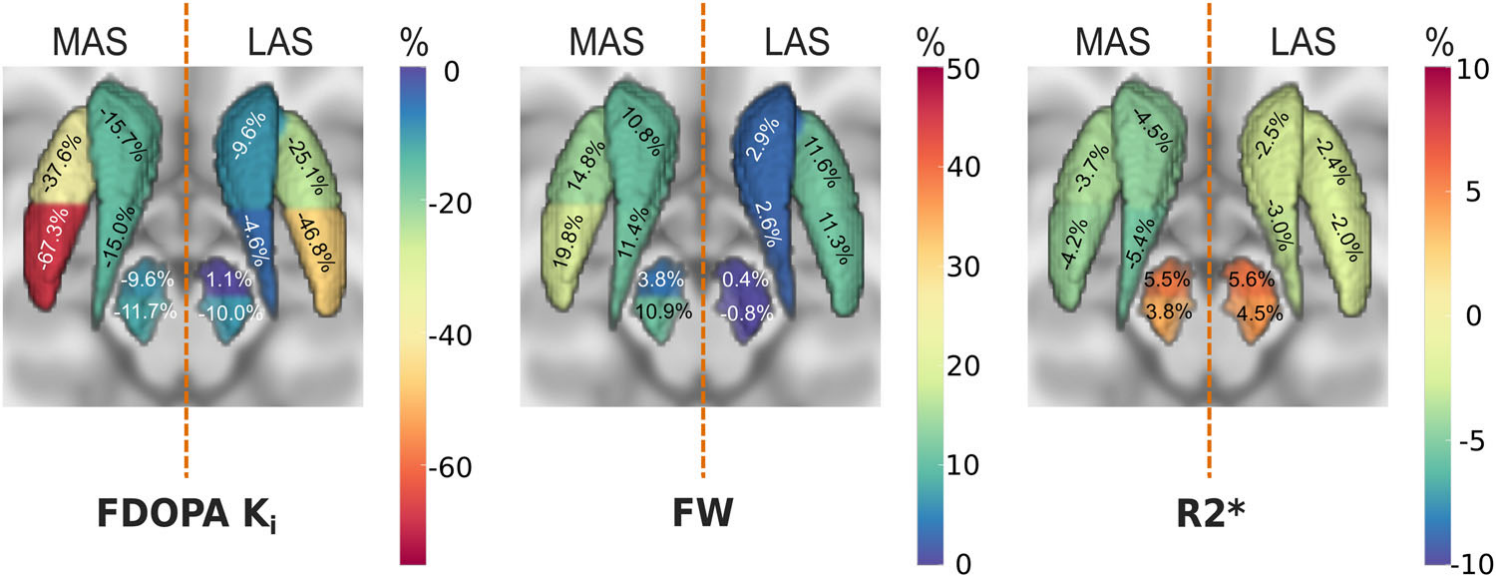
Nigrostriatal profile of change across imaging metrics. Percent of change for FDOPA, FW, and R2* in all nigrostriatal divisions between healthy subjects (HS) and PD patients. More affected and less affected sides (MAS/LAS) were compared to the dominant and non-dominant sides respectively. Representation is visualized in an axial view with the striatum and the SNc represented as a surface mesh. Percentages are highlighted by color-coding. Positive and negative changes represent increments and reductions for the metrics in PD patients with respect to the HS cohort.

FW was significantly higher within several brain regions in the MAS in PD patients compared to healthy subjects (HS) (Table 1 and Fig. 1). This was most pronounced in the posterior putamen (20%, *d* = 0.96, *P* < 0.01, *K* > 0.5) but was also noticeable in the anterior putamen (15%, *d* = 0.76, *P* < 0.05, *K* > 0.5), both caudate divisions (11%, *d* = 0.75, *P* < 0.05, *K* > 0.5), and posterolateral SNc (11%, *d* = 0.80, *P* < 0.05, *K* > 0.5). No significant changes were found in any region of the LAS. Interestingly, the pattern of FW increments paralleled the topography of FDOPA *K*_i_ reduction in the striatum and SNc (Fig. 2).

R2* was significantly decreased in the posterior caudate of the MAS, showing a modest but significant decrease in PD compared to HS (5.5%, *d* = 0.98, *P* < 0.05, *K* > 1). Further, we found R2* alterations that approached significance in opposite directions for the anterior caudate (4.5%, *d* = 0.67, *P* < 0.10, *K* > 0) and the anteromedial SNc (5.5%, *d* = 0.62, *P* < 0.10, *K* > 0), with decreased and increased R2* values in PD, respectively. See Supplementary Table 1 for a quantitative listing of FW and R2* measures.

### Multimodal associations within the nigrostriatal system

**Fig. 3:**
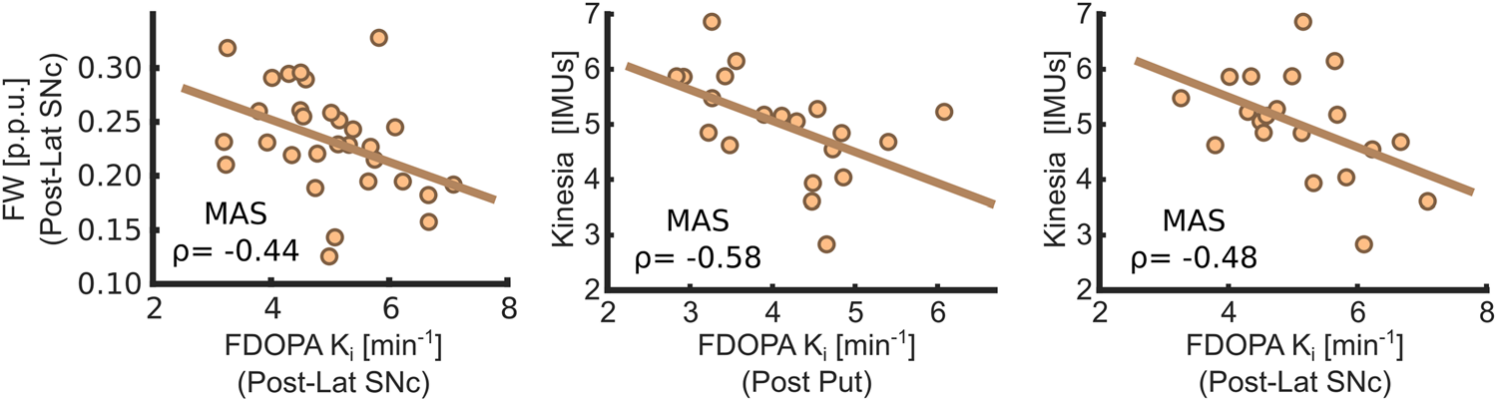
Scatter plots between imaging metrics and clinical severity. These scatter plots represent the correlation between FDOPA *K*_i_ and FW in the posterolateral SNc, and between FDOPA *K*_i_ in the posterior putamen and posterolateral SNc with the bradykinesia quantitative scores (Kinesia evaluation). The strength of these correlations (Spearman’s *ρ*) is reported within each scatter plot.

FW and FDOPA *K*_i_ in the posterolateral SNc of the MAS displayed a significant negative correlation (*ρ* = −0.44, *P* < 0.05) in the PD group, indicating that higher values of FW were associated with lower dopaminergic activity (Fig. 3). This correlation was, however, not found for the striatal regions in either the putamen or the caudate.

Interestingly, FDOPA activity for the PD group demonstrated a nigrostriatal segregation favoring correlations with the putamen for the posterolateral SNc division (*P* < 0.01) and with the caudate for the anteromedial SNc division (*P* < 0.05). This is indicative of a higher FDOPA deficit in the posterolateral SNc co-occurring with a greater deficit in the putamen.

Finally, R2* measures were significantly positively correlated between SNc and striatum but showed no specificity for segregated subcircuits as in FDOPA. FW values displayed no significant correlations between these regions. An overview of all significant correlations between imaging metrics can be found in Supplementary Fig. 1.

### Imaging-clinical associations

Bradykinesia quantitative scores showed a significant negative correlation with FDOPA *K*_i_ in the MAS (Fig. 3). Thus, aggregate bradykinesia scores exhibited moderate-to-strong correlations with FDOPA *K*_i_ in all striatal regions of interest (ROI) and in the posterolateral SNc (*P* < 0.05) and this motor quantitative assessment. FW and R2* showed no significant association with clinical motor impairment.

## Discussion

We have assessed FDOPA *K*_i_ and FW in several regions of the nigrostriatal system to show that microstructural alterations mirror the spatial pattern of dopaminergic loss within the striatum and SNc early in the evolution of PD. These changes are restricted to the brain hemisphere contralateral to the clinically symptomatic side. Thus, the contralateral post-commissural putamen and posterolateral SNc were the most altered regions in both imaging techniques. Further, we found a direct correlation between microstructural integrity and dopaminergic activity in the posterolateral SNc.

In line with previous PET studies, the FDOPA *K*_i_ decline in the putamen followed a marked posterior-to-anterior gradient of degeneration^2,6,7,31^. FDOPA *K*_i_ loss was also significant in the caudate nucleus, but to a lesser magnitude, with a relatively homogeneous loss across the structure^32,33^. Decreased FDOPA *K*_i_ values were also shown bilaterally in the SNc. A mild dopaminergic decline has been described previously in the SNc using dopamine active transporter^34–36^, vesicular monoamine transporter type 2^31,37,38^, or FDOPA radioligands^39,40^. Here we provide further details by splitting the SNc into anteromedial and posterolateral tiers. There is a greater loss in the posterolateral SNc, which agrees with common understanding of SNc neuronal vulnerability in PD. The posterolateral SNc includes the nigrosome-1, a substructure placed in the ventrolateral tier of this nucleus that is known to be affected in the earliest stage of the disease^41^. Since neurons of the posterolateral SNc project to the posterior putamen^42^, this fits well with the striatal pattern of depletion. These findings confirm in vivo the concept of selective vulnerability of the posterolateral nigroputaminal projection at the onset of PD^5,43^.

Dopaminergic activity was significantly associated between nigrostriatal regions. These associations were enhanced in PD as compared to HS, a result that might be explained by the global impact of disease onset in the nigrostriatal circuit. Interestingly, in PD there was a nigrostriatal segregation favoring dopaminergic connectivity between the anteromedial SNc and caudate on one side and the posterolateral SNc and the putamen on the other, further indicating a pattern of selective vulnerability for nigrostriatal dopaminergic degeneration^44,45^.

FW analysis revealed significant nigrostriatal alterations in the most affected hemisphere. In line with previous studies, our PD cohort exhibited an FW increment in the posterolateral SNc^18–21,46–48^, a feature that was correlated with FDOPA *K*_i_ reduction in the same region. Accordingly, this increment in nigral FW is congruent with the observed dopaminergic decline^5,41,49^, presumably reflecting neuronal loss. In contrast, no correlation was found between nigral FW increment and striatal dopaminergic denervation, which may be a consequence of the very short time since diagnosis (<12 months), large redundancy of striatal dopaminergic innervation, and compensatory capacity, particularly evident in mild PD patients.

There also was a widespread increment in striatal FW in our PD cohort, which exhibited a similar posterior-to-anterior gradient as for FDOPA loss, i.e., the post-commissural putamen was the most affected region. Few studies have previously examined striatal alterations in PD with the bi-compartmental diffusion model, and none of them reported significant findings^19,21,47^. Nevertheless, there is clear evidence regarding this occurrence using either classical DTI^50–52^ or other multicompartmental models^53,54^. We show here that FW is sensitive to microstructural alterations within the striatum, but restricted to the symptomatic side, so it could be considered as a potential biomarker for clinical onset and/or progression in PD. It is noteworthy, that striatal cell loss is not a pathological feature of PD^55^, but a marked reduction in axonal arborization and dendritic pruning are directly associated with dopaminergic denervation. Accordingly, the diffusion of water molecules restricted by tissue architecture is facilitated, perhaps explaining our finding of increased FW in early PD^56,57^. On the other hand, the absence of significant microstructural alterations on the LAS stands in sharp contrast to the extensive dopaminergic hypofunction seen there, indicating a possible early disturbance in the dopaminergic activity prior to axonal degeneration or neuronal loss. Indeed, it has already been suggested that SNc neurons and dopaminergic fibers may survive for some time after losing their dopaminergic function, perhaps being able to recover this capacity if treated early enough^16^. Further investigation needs to be done, but if confirmed, this feature could potentially unlock new opportunities for novel restorative therapies, further emphasizing the relevance of an early detection of the disease.

Although there was no direct correlation between striatal FW and FDOPA *K*_i_, the highest increment in FW matched the most denervated region, i.e., the posterior putamen, which suggests a relationship between these two processes. Previous evidence points to two principal structural mechanisms that may be linked to striatal denervation to explain this relationship: (1) a loss of synaptic terminals and dendritic arborization^58^; and (2) a significant reduction of dendritic spines^59^. Both alterations might well facilitate water diffusion and therefore lead to higher FW values. This is a potentially remarkable aspect that should be validated first experimentally and, secondly, throughout disease progression in PD patients.

Previously, several studies have detected iron accumulation using iron-sensitive MRI techniques^27,29,46,60,61^. In this cohort, we failed to demonstrate significant iron alterations within the SNc, but we did encounter a tendency towards increased iron in the anteromedial SNc. We don´t think the discrepancy with previous results stems from technical factors. We believe it is probably related to the limited clinical severity of our patients who, in addition, were drug naive^62^. Thus, reduced cell loss and lack of dopaminergic metabolic activation (by levodopa treatment) might explain the insufficient statistical evidence of iron abnormalities in the SNc.

The present study had several limitations. The number of patients in our cohort is moderate in size. This group of de novo PD patients had predominant motor manifestations, with very asymmetric and focal presentation^6^. Importantly, this allowed us to test the validity of our biomarkers in a minimally symptomatic hemisphere. Based on disease characteristics, we should also consider the relatively younger age of our PD cohort. Several studies have described disease-specific traits as a function of the age at PD onset^52,63,64^. Accordingly, we cannot exclude that some of the differences relative to controls might be specific to young PD onset. These differences might explain the lack of findings in the literature when using FW to assess microstructural integrity in the striatum^19,47^ or our inability to detect significant alterations in the SNc when assessing iron content. In addition, the possibility that some of the patients included here would actually suffer from a different neurodegenerative condition, principally MSA, is acknowledged. This is always a possibility in studies of recently diagnosed patients. We limited largely this false positive inclusion by studying only patients with very focal motor onset including resting tremor in a high proportion, and a correlatively focal dopaminergic loss in the posterior putamen characteristic of early PD^6,7^. The median age of onset was also lower than is typical for MSA, PSP, etc., and they exhibited no atypical symptomatology (i.e., blood pressure hypotension, urinary or erectile dysfunction, eye-movement limitation, reflex myoclonus, etc.). Finally, the now available evolution (after 4-5 years of follow-up) has not revealed any atypical manifestation in any patient. Another limitation might be the feasibility of FDOPA as a precise marker of nigrostriatal dopaminergic denervation in early PD. FDOPA activity might be upregulated in early-stage PD^3^. At the time of the study, other radiotracers such as vesicular monoamine transporter type 2, which might provide better sensitivity in early stages of PD and could lead to more robust intermodal correlations, were not available. Nevertheless, FDOPA has previously demonstrated a significant capacity to define precise topography of striatal dopaminergic integrity loss^7^. This, in addition to its direct relationship to clinical impairment, seems sufficient to accept its use as an adequate marker of dopaminergic integrity in early-stage PD. Finally, SNc delineation with MRI is challenging. Defining SNc boundaries with respect to the SNr or the subthalamic nucleus is extremely difficult if not impossible with the currently available neuroimaging methodology. Multiple approaches have been proposed based on either manual or semiautomatic methods using different MRI sequences, but no gold-standard exists so far^22,52,65^. Here, we applied a fully automated atlas-based division using anatomical landmarks as reference (red nucleus), which provided a robust inter-subject allocation and minimized observer bias. Further studies should aim to demonstrate the impact that specific parcellation strategies have on these biomarkers.

In conclusion, we show here that early PD is associated with a specific anatomical pattern of dopaminergic and microstructural changes in the nigrostriatal system as evaluated by FDOPA PET uptake and MRI free-water imaging. In comparison to the SNc, the striatal putamen was significantly more affected in both FDOPA and FW imaging. Thus, even though the percentage change in the posterolateral SNc was comparable in magnitude between dopaminergic and microstructural biomarkers (-11.7% of FDOPA *K*_i_ vs +10.9% in FW), they differed significantly in the putamen (-67% vs +20%). This nigrostriatal asymmetry for dopaminergic denervation and microstructural abnormalities might indicate greater pathological alteration of the striatum (the posterior putamen mainly) versus the SNc. In addition, the lack of significant changes in the microstructure of the minimally symptomatic side along with a net decrease in FDOPA levels suggests a predominant and initial loss of striatal dopaminergic innervation preceding major SNc neuronal loss. Furthermore, although non-statistically significant (*P* = 0.11), there were some hints pointing to minor microstructural alterations in the putamen (~11.5% in FW) of the LAS, whereas no sign of change was observed in the SNc (~0). Although we cannot rule out the possibility that neurodegeneration may begin simultaneously in both the striatum and the SNc, these results altogether support the contention of a relatively focal axo-synaptic onset of neurodegeneration in PD^10–16^. Further multimodal studies are needed to conclusively verify the focal pathological onset of PD in the putamen, but as a corollary, our findings would suggest the convenience of considering the posterior putamen as the primary target for putative neurorestorative therapies^66–68^.

## Methods

### Study participants

Thirty de novo PD patients and twenty HS matched for age, sex, and handedness were included in this study. Participants were enrolled between June 2016 and October 2020 at the University Hospital HM Puerta del Sur (Móstoles, Spain). The inclusion criteria for the PD group were: de novo patients with less than 12 months of disease progression after diagnosis and with unilateral motor impairment, i.e., signs were predominantly confined to one hemibody. The diagnosis of PD was made according to the UK Brain Bank Clinical Criteria by three neurologists specialized in movement disorders (ASF, MHGM, JAO). Neurological examinations included the Movement Disorders Society Unified Parkinson’s Disease Rating Scale Part-III (MDS-UPDRS-III). Bradykinesia tests were complemented with quantitative assessments using inertial measurement units (IMUs) (Kinesia™ One system; Great Lakes Neurotechnologies Inc, Cleveland, OH)^69^. Further details about the neurological examinations can be found in Monje et al. *(Mov. Disord., 2021)*^6^. The study protocol was approved by the Ethics Committee of HM Hospitales (protocol number: 16.10.0993-GHM). All participants provided written informed consent in accordance with local regulations. Demographic features are described in Table 2.

**Table 2:** Demographic data.

	HS (*n* = 20)	PD (*n* = 30)	*P* value (PD vs HS)
Age	50.35 ± 11.02	54.93 ± 8.72	0.17
Sex (men/women)	10/10	18/12	0.50
Hand dominance (R/L)	18/2	26/4	0.67
Side of onset (R/L)	-	20/10	-
Time since diagnosis (months)	-	7.15 ± 5.63	-
Time from onset (months)	-	13.81 ± 8.85	-
Total MDS-UPDRS III	-	19.97 ± 9.08	-
MAS - MDS-UPDRS III	-	11.87 ± 4.37	-
LAS - MDS-UPDRS III	-	2.57 ± 3.57	-
Total Kinesia	-	9.50 ± 2.19	-
MAS - Kinesia	-	5.17 ± 1.20	-
LAS - Kinesia	-	4.34 ± 1.17	-

Value ± standard deviation.

*MAS* more affected side, *LAS* less affected side.

### Imaging protocol

Whole-brain MRI and FDOPA PET scans were collected using a hybrid 3 T mMR-Biograph system (Siemens Healthcare, Erlangen, Germany). PET sessions were carried out with subjects at rest and after at least 6-hours of fasting. Following an intravenous injection of ~5 mCi of FDOPA, PET data were acquired in list-mode for 90 min. Images were reconstructed with an ordered subset-expectation maximization algorithm and were smoothed by applying a 3D isotropic Gaussian kernel with 4 mm full width at half maximum. Scans were then corrected for attenuation using a 4-compartment MR-based map derived from a dual-echo Dixon-based sequence (TR: 3.6 ms; TE: [1.23,2.46] ms), that incorporates bone information using a model-based segmentation algorithm^70^. Twenty-two activity timeframes were reconstructed (10 frames of 90 s, 9 frames of 300 s, and 3 frames of 600 s) with an effective resolution of 2.09 × 2.09 × 2.03 mm^3^. Simultaneously to the PET acquisition, the MRI protocol included: (a) A 3D T1-weighted (T1w) image acquired using a magnetization-prepared rapid acquisition gradient echo (M-PRAGE) sequence (TR/TE: 2300/3.34 ms; flip angle: 12°; FoV: 256 mm; in-plane matrix: 256 × 256; in-plane resolution: 1 × 1 mm^2^; slice thickness: 1mm; 176 sagittal slices); (b) Single-shell DWI based on a single-shot 2D spin-echo sequence (*b* value = 1000 s/mm^2^; 64 gradient directions with anterior-to-posterior phase encoding; TR/TE: 10000/102 ms; flip angle: 90°; FoV: 256 mm; in-plane matrix: 128 × 128; in-plane resolution: 2 × 2 mm^2^; slice thickness: 2 mm; 24 slices), with 4 *b* value = 0 s/mm^2^ scans at the beginning of the sequence. Four additional *b* value = 0 s/mm^2^ images but with reverse-phase encoding (posterior-to-anterior) direction were also acquired in order to correct EPI distortions; (c) A 3D multi-echo gradient echo (multi-GRE) sequence recorded with the following specifications: TR: 60 ms; 10 echoes; TEs: [4.36:46.12] ms; inter-echo spacing: 4.64 ms; flip angle: 20°; FoV: 230 mm; in-plane matrix: 256 × 256; in-plane resolution: 0.9 × 0.9 mm^2^; slice thickness: 2 mm; 60 slices.

### Image processing

DICOM data were converted to NIFTI format using the dcm2niix package^71^. T1w scans were corrected for intensity bias^72^ and skull-stripped with BET-FSL (FMRIB Software Library) v6.0^73,74^. Then, anatomical images were registered nonlinearly to the ICBM-152 2009c Nonlinear Symmetric (ICBM-152) template computing an affine transformation followed by a diffeomorphic symmetric normalization^75^. Magnitude images from multi-GRE acquisitions were used to generate iron-sensitive maps. Relaxometry R2* maps were reconstructed by applying nonlinear fitting of the complex monoexponential equation with the autoregressive algorithm^76^ implemented in the MEDI toolbox^77^. R2* maps were rigidly registered to T1w space using as reference the magnitude image of the first echo. DWI pre-processing included data denoising^78^, motion, susceptibility-induced, and eddy current-induced distortion corrections (topup and eddy in FSL)^79–81^, and bias field correction^72^. Non-brain tissue was removed with BET-FSL before DWI pre-processing. Corrected DWI scans were rigidly registered to T1w space using the first *b* = 0 s/mm^2^ image as reference. FW maps were estimated with the Dipy library^82^ by fitting the bi-tensor model with the regularized gradient descent method and applying the hybrid initialization strategy described by Parker et al. (*PLoS One*, 2020)^83^. To reduce motion effects during PET exams, activity frames were realigned within subjects applying a rigid-body transformation with the Mc-FLIRT tool in FSL^84^. Voxel-based FDOPA uptake rate maps (described as constant *K*_i_ [min^−1^]) were estimated using the Patlak graphical method^85^ and taking the average time-activity curve from an occipital lobe mask as a reference. Intensity bias correction and coregistration steps were conducted with ANTs (Advanced Normalization Tools) v2.3.1^86^.

### Regions of interest

**Fig. 4:**
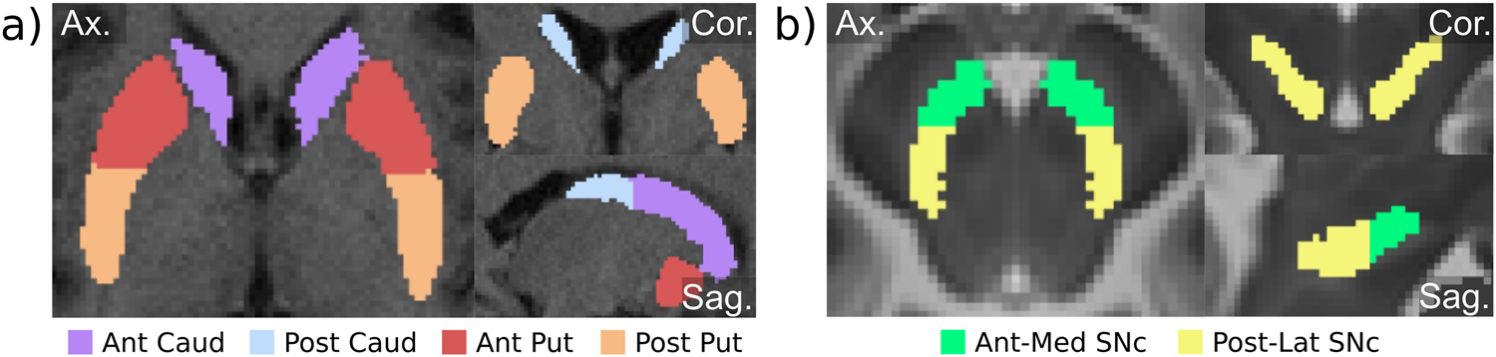
ROI definition scheme. **a** Anterior and posterior divisions of the striatum in both caudate and putamen structures in the T1w native space in one representative subject. The division was made with respect to the anterior commissure. **b** Anteromedial and posterolateral divisions of the substantia nigra pars compacta (SNc) with respect to the centroid of the red nucleus overlaid onto a T2w in standard ICBM-152 2009c space.

Several ROIs were defined across the SNc and striatum. Segmentation of the SNc was extracted from a reference atlas^87^ (Prob. ≥ 0.001). The segmentation was partitioned into anteromedial and posterolateral divisions setting a vertical cutting plane across the centroid of the red nucleus (also extracted from the same atlas) (Fig. 4b). SNc ROIs were built in the ICBM-152 template space. To keep mask symmetry between hemibrains, the left mask was isolated and subsequently mirrored.

Whole-putamen and whole-caudate masks were segmented from structural T1w with the FMRIB integrated registration and segmentation tool (FIRST)^88^. Pre- and post-commissural divisions were defined by applying a cutting plane set across the anterior commissure (Fig. 4a). In order to avoid data blurring caused by spatial transformations between image spaces, ROIs were transformed to the native space of the imaging metrics (FW, R2*, and FDOPA *K*_i_) applying a nearest-neighbor interpolation strategy.

### Statistical analyses

Mean values were computed within ROIs, that were sorted according to the side of the predominance of motor signs (PD) or hand dominance (HS). Consequently, ROI data were divided into MAS and LAS and dominant and non-dominant sides (DS and nDS). Statistical comparisons between groups were carried out by applying the Mann–Whitney *U* tests separately for MAS (DS in HS) and LAS (nDS in HS). Statistical significance was set at *P* < 0.05. The Cohen *d* was used to assess effect size. Complementary to *P* values, Bayes factors (BF) were computed and reported on a logarithmic scale (*K* *=* *log*_*10*_*(BF)*^89^) to improve text readability. Logarithmic BFs were interpreted as follows 0 < *K* ≤ 0.5 (barely worth mentioning); 0.5 < *K* ≤ 1 (substantial); 1 < *K* ≤ 2 (strong); *K* > 2 (decisive)^89^.

Non-parametric Spearman correlation analyses were performed to study the inter-regional interactions and the associations between imaging metrics. All correlations were tested independently for HS and PD groups. Results were adjusted for multiple comparisons using the Storey FDR method. Finally, imaging metrics were compared contralaterally with unilateral bradykinesia aggregated scores^6^. Classical and Bayesian statistics were respectively computed with MATLAB (The MathWorks Inc., United States) and JASP v.0.16.1 (JASP Team, 2022).

### Reporting summary

Further information on research design is available in the Nature Research Reporting Summary linked to this article.

### Supplementary information

Supplementary material

Reporting Summary

## Data Availability

The datasets generated during the current study are available in the Zenodo repository (https://zenodo.org/record/8367307).

## Code availability

Image processing packages are freely available in their respective websites: dcm2niix: https://github.com/rordenlab/dcm2niix; FSL v6.0: https://fsl.fmrib.ox.ac.uk/fsl/fslwiki/FSL; MEDI Toolbox: https://pre.weill.cornell.edu/mri/pages/qsm.html; Dipy: https://dipy.org; ANTs: http://stnava.github.io/ANTs/. In-house MATLAB codes developed for the statistical analyses are available to any qualified researcher from the corresponding author.
