# Supplementary material for: Dopaminergic denervation and associated MRI microstructural changes in the nigrostriatal projection in early Parkinson’s disease patients
Source: NPJ Parkinsons Dis. 2023 Oct 19;9:144. doi: 10.1038/s41531-023-00586-x (PMC10584921; doi:10.1038/s41531-023-00586-x)
Supplement: Supplementary file 1 — Supplementary material [file 41531_2023_586_MOESM1_ESM.docx]

## Supplementary Figures


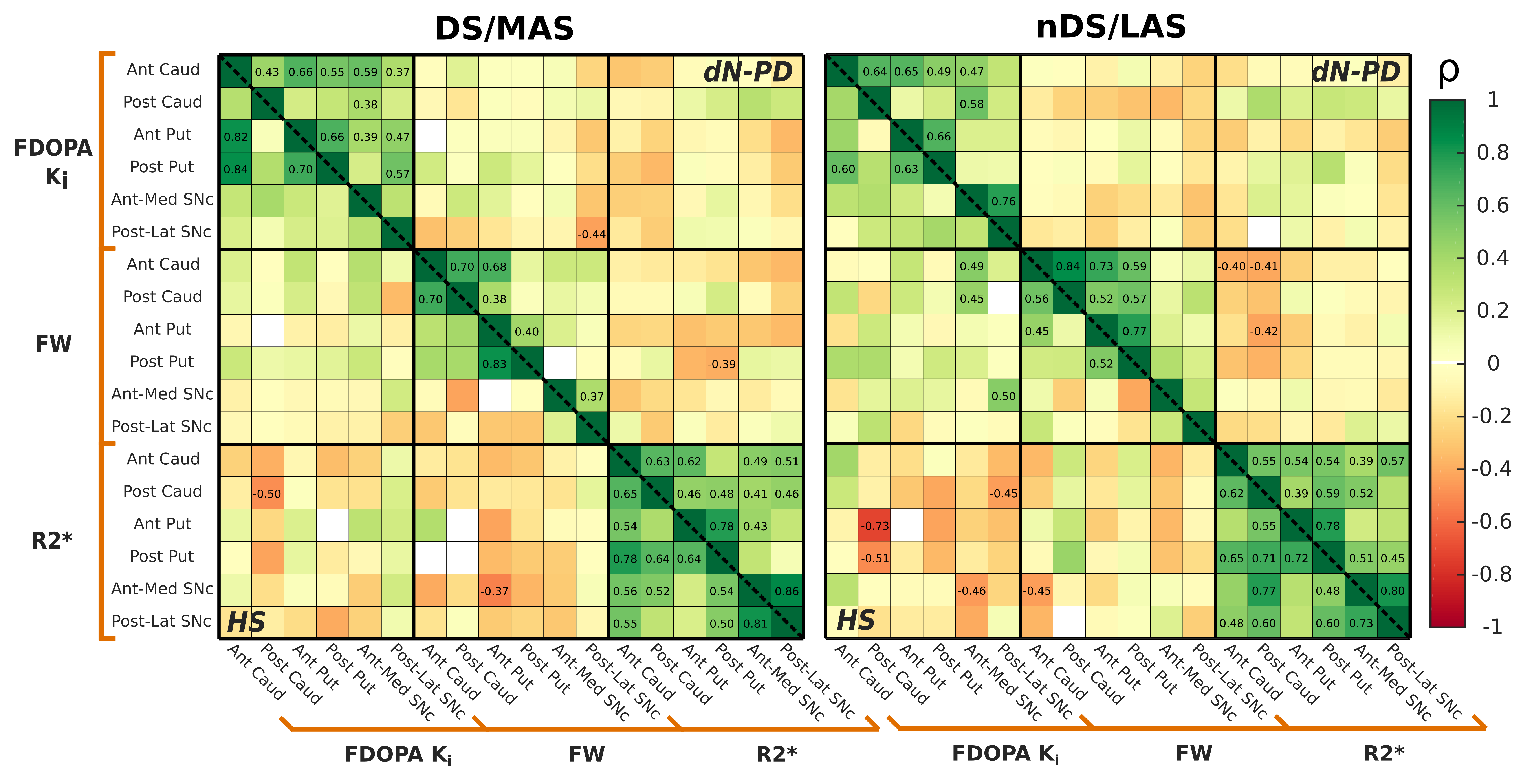


Supplementary Figure 1 - Correlation matrices between imaging metrics. These matrices displayed the associations between imaging metrics within caudate, putamen, and SNc ROIs. The correlations found for the PD/HS group have been displayed by the upper/lower triangular matrix. MAS and LAS respectively represent the more and less affected sides in the PD group. Analogously, DS and nDS denote the dominant and non-dominant sides of the HS group. The strength of the interactions (Spearman ρ) has been color-coded: greens are assigned to direct associations and oranges/reds to inverse ones. Correlation values are reported only for interactions that have reached statistical significance (P ≤ 0.05).

## Supplementary Tables

Supplementary Table 1 – Mean values within ROIs.

|  | ***ROI*** | ***HS (DS)*** | ***dN-PD (MAS)*** | ***% Diff***  ***(MAS vs DS)*** | ***HS (nDS)*** | ***dN-PD (LAS)*** | ***% Diff***  ***(LAS vs nDS)*** |
| --- | --- | --- | --- | --- | --- | --- | --- |
| ***FDOPA***  **[min^-1^]** | ***Ant Caud*** | 12.21±0.79 | 10.29±0.98 | -15.71 | 12.19±0.91 | 11.03±0.77 | -9.55 |
|  | ***Post Caud*** | 7.62±1.01 | 6.47±0.75 | -15.07 | 7.22±0.87 | 6.88±0.85 | -4.63 |
|  | ***Ant Put*** | 12.88±0.75 | 8.04±0.84 | -37.58 | 13.27±0.78 | 9.94±1.02 | -25.06 |
|  | ***Post Put*** | 12.27±0.99 | 4.02±0.58 | -67.29 | 12.02±0.75 | 6.40±0.93 | -46.76 |
|  | ***Ant-Med SNc*** | 5.86±0.59 | 5.30±0.57 | -9.59 | 5.82±0.80 | 5.88±1.00 | 1.06 |
|  | ***Post-Lat SNc*** | 5.63±0.57 | 4.98±0.68 | -11.68 | 5.90±0.76 | 5.30±0.67 | -10.04 |
| ***FW***  **[p.p.u.]** | ***Ant Caud*** | 0.18±0.02 | 0.20±0.03 | 10.82 | 0.20±0.02 | 0.21±0.04 | 2.92 |
|  | ***Post Caud*** | 0.24±0.03 | 0.27±0.04 | 11.36 | 0.26±0.03 | 0.26±0.04 | 2.62 |
|  | ***Ant Put*** | 0.13±0.02 | 0.14±0.03 | 14.83 | 0.12±0.02 | 0.14±0.02 | 11.61 |
|  | ***Post Put*** | 0.11±0.02 | 0.13±0.02 | 19.83 | 0.12±0.02 | 0.13±0.03 | 11.32 |
|  | ***Ant-Med SNc*** | 0.29±0.05 | 0.30±0.05 | 3.83 | 0.30±0.05 | 0.30±0.04 | 0.38 |
|  | ***Post-Lat SNc*** | 0.21±0.03 | 0.23±0.03 | 10.93 | 0.23±0.04 | 0.22±0.03 | -0.76 |
| ***R2** [s^-1^]** | ***Ant Caud*** | 22.32±1.76 | 21.32±1.24 | -4.50 | 22.22±2.06 | 21.66±1.40 | -2.50 |
|  | ***Post Caud*** | 22.65±1.39 | 21.42±1.13 | -5.42 | 22.35±1.27 | 21.68±1.09 | -2.98 |
|  | ***Ant Put*** | 25.69±1.89 | 24.73±1.29 | -3.74 | 25.24±1.97 | 24.63±1.11 | -2.42 |
|  | ***Post Put*** | 26.18±2.51 | 25.09±2.00 | -4.16 | 26.41±2.98 | 25.88±2.35 | -2.01 |
|  | ***Ant-Med SNc*** | 32.62±2.45 | 34.40±3.13 | 5.47 | 32.33±3.41 | 34.14±4.22 | 5.60 |
|  | ***Post-Lat SNc*** | 25.20±2.29 | 26.15±2.79 | 3.77 | 24.19±2.36 | 25.27±2.91 | 4.47 |
| **Mean value ± standard deviation.**  **% Diff (A vs B) = Difference in percentage between A and B mean values. The sign represents the direction of the effect, i.e., for A vs B, % Diff < 0 means that A < B and vice versa.** | | | | | | | |
