# Supplementary material for: Dopaminergic denervation and associated MRI microstructural changes in the nigrostriatal projection in early Parkinson’s disease patients
Source: NPJ Parkinsons Dis. 2023 Oct 19;9:144. doi: 10.1038/s41531-023-00586-x (PMC10584921; doi:10.1038/s41531-023-00586-x)
Supplement: Supplementary file 2 — Reporting Summary [file 41531_2023_586_MOESM2_ESM.pdf]

nature portfolio

Corresponding author(s): Dr José (A.) Pineda-Pardo

Last updated by author(s): Sep 13, 2023

Reporting Summary

Nature Portfolio wishes to improve the reproducibility of the work that we publish. This form provides structure for consistency and transparency in reporting. For further information on Nature Portfolio policies, see our Editorial Policies and the Editorial Policy Checklist.

Statistics

For all statistical analyses, confirm that the following items are present in the figure legend, table legend, main text, or Methods section.

n/a	Confirmed	
☐	☒	The exact sample size (*n*) for each experimental group/condition, given as a discrete number and unit of measurement
☒	☐	A statement on whether measurements were taken from distinct samples or whether the same sample was measured repeatedly
☐	☒	The statistical test(s) used AND whether they are one- or two-sided<br>*Only common tests should be described solely by name; describe more complex techniques in the Methods section.*
☐	☒	A description of all covariates tested
☐	☒	A description of any assumptions or corrections, such as tests of normality and adjustment for multiple comparisons
☐	☒	A full description of the statistical parameters including central tendency (e.g. means) or other basic estimates (e.g. regression coefficient) AND variation (e.g. standard deviation) or associated estimates of uncertainty (e.g. confidence intervals)
☐	☒	For null hypothesis testing, the test statistic (e.g. *F*, *t*, *r*) with confidence intervals, effect sizes, degrees of freedom and *P* value noted<br>*Give P values as exact values whenever suitable.*
☒	☐	For Bayesian analysis, information on the choice of priors and Markov chain Monte Carlo settings
☒	☐	For hierarchical and complex designs, identification of the appropriate level for tests and full reporting of outcomes
☐	☒	Estimates of effect sizes (e.g. Cohen's *d*, Pearson's *r*), indicating how they were calculated

Our web collection on statistics for biologists contains articles on many of the points above.

Software and code

Policy information about availability of computer code

Data collection	No software was used
Data analysis	MATLAB R2016b, JASP v.0.16.1, dcm2niix, FSL v6.0, MEDI Toolbox, Dipy v1.6.0.and ANTs v2.3.1

For manuscripts utilizing custom algorithms or software that are central to the research but not yet described in published literature, software must be made available to editors and reviewers. We strongly encourage code deposition in a community repository (e.g. GitHub). See the Nature Portfolio guidelines for submitting code & software for further information.

Data

Policy information about availability of data

All manuscripts must include a data availability statement. This statement should provide the following information, where applicable:

- Accession codes, unique identifiers, or web links for publicly available datasets
- A description of any restrictions on data availability
- For clinical datasets or third party data, please ensure that the statement adheres to our policy

The data supporting these study findings are available from the corresponding author upon request by any qualified investigator, preferably under a collaborative agreement.

Research involving human participants, their data, or biological material

Policy information about studies with human participants or human data. See also policy information about sex, gender (identity/presentation), and sexual orientation and race, ethnicity and racism.

Reporting on sex and gender	Gender data were not gathered and solely biological sex information was obtained. Both study groups were balanced in terms of sex, with no explicit sex-based comparisons performed. Sex distribution was 10/10 males/females in the HS group and 18/12 in the PD group. Despite investigating sex-specific differences related to FDOPA, FW, or R2* is an intriguing avenue for future research, this was out of the scope of the present study. In addition, our sample size might not be large enough to effectively address such questions
Reporting on race, ethnicity, or other socially relevant groupings	Race, ethnicity or social group information was not collected in this study.
Population characteristics	This study comprised 30 de novo PD patients and 20 HS, matched for age, sex and handedness. The mean age was 50.35 ±11.02 for the HS group and 54.93±8.72 for the PD group. Our cohorts consisted of an equal distribution of 10 males and 10 females in the HS group and 18 males and 12 females in the PD group. Additionally, both groups predominantly consisted of right-handed individuals (18/20 in the HS group and 26/30 in the PD group). In the PD group, all patients were clinically asymmetric, with 20 patients being more affected in the right hemibody, and 10 in the left hemibody.
Recruitment	We recruited a consecutive cohort of drug-naive PD patients and HS as part of a prospective 2-year longitudinal study (Monje et al 2021, Movement Disorders; Pineda-Pardo et al 2022, Brain) between June 2016 and October 2020 at the University Hospital HM Puerta del Sur (Móstoles, Spain). The inclusion criteria for the PD group were: de novo patients with less than 12 months of disease progression after diagnosis and with unilateral motor impairment. The diagnosis of PD was made according to the UK Brain Bank Clinical Criteria. Healthy subjects were recruited from several groups including PD patient relatives, hospital healthy visitors, research volunteers or university members.
Ethics oversight	HM Hospitales (protocol number: 16.10.0993-GHM)

Note that full information on the approval of the study protocol must also be provided in the manuscript.

Field-specific reporting

Please select the one below that is the best fit for your research. If you are not sure, read the appropriate sections before making your selection.

☒ Life sciences ☐ Behavioural & social sciences ☐ Ecological, evolutionary & environmental sciences

For a reference copy of the document with all sections, see nature.com/documents/nr-reporting-summary-flat.pdf

Life sciences study design

All studies must disclose on these points even when the disclosure is negative.

Sample size	We did not employ any specific statistical methods to predefine the sample size. The sample size, N=20HS/30PD, is consistent with that of other imaging studies investigating neurodegeneration in Parkinson's disease.
Data exclusions	No subjects were excluded.
Replication	We did not undertake specific measures to assess the reproducibility of our findings.
Randomization	Allocation was not random. PD patients were assigned according to the inclusion criteria (see above) and HS were PD patient relatives or random volunteers matched for age, sex and handedness with the PD group.
Blinding	This study employed a multimodal crossover design involving both PD and HS. One of the primary modalities used was FDOPA PET imaging, which limited our ability to conduct a blinded comparison between the two groups.

Reporting for specific materials, systems and methods

We require information from authors about some types of materials, experimental systems and methods used in many studies. Here, indicate whether each material, system or method listed is relevant to your study. If you are not sure if a list item applies to your research, read the appropriate section before selecting a response.

Materials & experimental systems

n/a	Involved in the study
☒	☐ Antibodies
☒	☐ Eukaryotic cell lines
☒	☐ Palaeontology and archaeology
☒	☐ Animals and other organisms
☐	☒ Clinical data
☒	☐ Dual use research of concern
☒	☐ Plants

Methods

n/a	Involved in the study
☒	☐ ChIP-seq
☒	☐ Flow cytometry
☐	☒ MRI-based neuroimaging

Clinical data

Policy information about clinical studies

All manuscripts should comply with the ICMJE guidelines for publication of clinical research and a completed CONSORT checklist must be included with all submissions.

Clinical trial registration: This study was not part of a clinical trial

Study protocol: This study was not part of a clinical trial

Data collection: Patient recruitment took place between June 2016 and October 2020 at the University Hospital HM Puerta del Sur (Móstoles, Spain).

Outcomes: We aimed to study structural and metabolic alterations within the nigrostriatal system at the clinical onset of motor features in a group of de novo PD patients. We assessed the degree and spatial pattern of dopaminergic denervation (FDOPA), microstructural integrity (FW), and iron accumulation (R2*) within several regions of the nigrostriatal system, including spatial divisions of the SNc and striatum.

Magnetic resonance imaging

Experimental design

Design type: No fMRI was used in this study

Design specifications: No fMRI was used in this study

Behavioral performance measures: No behavioral performance measures were used

Acquisition

Imaging type(s): Structural MRI, quantitative MRI, diffusion MRI and PET.

Field strength: 3 T

Sequence & imaging parameters: (a) A 3D T1-weighted (T1w) image acquired using a magnetization-prepared rapid acquisition gradient echo (M-PRAGE) sequence (TR/TE: 2300/3.34ms; flip angle: 12º; FoV: 256mm; in-plane matrix: 256x256; in-plane resolution: 1x1mm2; slice thickness: 1mm; 176 sagittal slices); (b) A 3D multi-echo gradient echo sequence recorded with the following specifications: TR: 60ms; 10 echoes; TEs: [4.36:46.12] ms; inter-echo spacing: 4.64 ms; flip angle: 20º; FoV: 230mm; in-plane matrix: 256x256; in-plane resolution: 0.9x0.9mm2; slice thickness: 2mm; 60 slices.

Area of acquisition: Whole brain scan

Diffusion MRI: ☒ Used ☐ Not used

Parameters: Single shell, b-value = 1000s/mm2; 64 gradient directions with anterior-to-posterior phase encoding; TR/TE: 10000/102ms; flip angle: 90º; FoV: 256mm; in-plane matrix: 128x128; in-plane resolution: 2x2mm2; slice thickness: 2mm; 24 slices; 4 b-value = 0s/mm2 scans at the beginning of the sequence

Preprocessing

Preprocessing software: dcm2niix, FSL v6.0, MEDI Toolbox, Dipy v1.6.0., MATLAB R2016b, ANTs v2.3.1

Normalization: Normalization transformations were calculated using ANTs v2.3.1. Nevertheless, all analyses were conducted within the native space after transformation both atlas-based or T1w-based ROIs to this space.

Normalization template: ICBM-152 2009c Nonlinear Symmetric template

Noise and artifact removal: T1w bias correction in was perfomed using a N4 algorithm implemented in ANTs v2.3.1. DWI data was denoised using the overcomplete local PCA method. DWI artifacts were corrected with eddy and topup in FSL v6.0. Bias correction was applied using the previous N4 algorithm.

Volume censoring: No volume censoring was applied

Statistical modeling & inference

Model type and settings: Univariate non-parametric Mann Mann-Whitney U tests were used in both Classical and Bayesian Statistics

Effect(s) tested: The attempted comparisons involved evaluating differences between PD and HS, specifically assessing whether PD biomarker measurements were higher or lower than those in HS.

Specify type of analysis: ☐ Whole brain ☒ ROI-based ☐ Both

Anatomical location(s): Substantia nigra pars compacta, putamen and caudate

Statistic type for inference (See Eklund et al. 2016): Only ROI based comparisons were performed.

Correction: Significance after correlation analyses was corrected for multiple comparisons using FDR correction.

Models & analysis

n/a	Involved in the study
☒	☐ Functional and/or effective connectivity
☒	☐ Graph analysis
☒	☐ Multivariate modeling or predictive analysis
